# Transient axonal glycoprotein-1 (TAG-1) and laminin-α1 regulate dynamic growth cone behaviors and initial axon direction *in vivo*

**DOI:** 10.1186/1749-8104-3-6

**Published:** 2008-02-20

**Authors:** Marc A Wolman, Vinoth K Sittaramane, Jeffrey J Essner, H Joseph Yost, Anand Chandrasekhar, Mary C Halloran

**Affiliations:** 1 Departments of Zoology and Anatomy, and Neuroscience Training Program, University of Wisconsin, Madison, Wisconsin 53706, USA; 2 Department of Neurobiology and Anatomy, University of Utah School of Medicine, Salt Lake City, Utah 84112-5330, USA; 3 Division of Biological Sciences, University of Missouri, Columbia, Missouri 65211, USA; 4 Department of Cell and Developmental Biology, University of Pennsylvania Medical School, Philadelphia, PA 19104, USA; 5 Department of Genetics, Development and Cell Biology, Iowa State University, Ames, IA 50011, USA

## Abstract

**Background:**

How axon guidance signals regulate growth cone behavior and guidance decisions in the complex *in vivo *environment of the central nervous system is not well understood. We have taken advantage of the unique features of the zebrafish embryo to visualize dynamic growth cone behaviors and analyze guidance mechanisms of axons emerging from a central brain nucleus *in vivo*.

**Results:**

We investigated axons of the nucleus of the medial longitudinal fascicle (nucMLF), which are the first axons to extend in the zebrafish midbrain. Using *in vivo *time-lapse imaging, we show that both positive axon-axon interactions and guidance by surrounding tissue control initial nucMLF axon guidance. We further show that two guidance molecules, transient axonal glycoprotein-1 (TAG-1) and laminin-α1, are essential for the initial directional extension of nucMLF axons and their subsequent convergence into a tight fascicle. Fixed tissue analysis shows that TAG-1 knockdown causes errors in nucMLF axon pathfinding similar to those seen in a laminin-α1 mutant. However, *in vivo *time-lapse imaging reveals that while some defects in dynamic growth cone behavior are similar, there are also defects unique to the loss of each gene. Loss of either TAG-1 or laminin-α1 causes nucMLF axons to extend into surrounding tissue in incorrect directions and reduces axonal growth rate, resulting in stunted nucMLF axons that fail to extend beyond the hindbrain. However, defects in axon-axon interactions were found only after TAG-1 knockdown, while defects in initial nucMLF axon polarity and excessive branching of nucMLF axons occurred only in laminin-α1 mutants.

**Conclusion:**

These results demonstrate how two guidance cues, TAG-1 and laminin-α1, influence the behavior of growth cones during axon pathfinding *in vivo*. Our data suggest that TAG-1 functions to allow growth cones to sense environmental cues and mediates positive axon-axon interactions. Laminin-α1 does not regulate axon-axon interactions, but does influence neuronal polarity and directional guidance.

## Background

In the developing nervous system, axon pathway formation is governed by signals that steer the axonal growth cone in the appropriate direction. Although multiple guidance signals have been identified, our understanding of how specific growth cone behaviors and pathfinding steps are regulated *in vivo *is far from complete. We are investigating mechanisms controlling the pathfinding of axons extending from the nucleus of the medial longitudinal fascicle (nucMLF) in the zebrafish brain. The nucMLF neurons are the first neurons to differentiate and extend axons in the midbrain. They navigate uncharted territory and establish a major axon tract upon which multiple later-growing axons extend. We have focused particularly on the initial pathfinding steps that nucMLF axons must make: emergence from the cell body with correct polarity, extension in the correct direction, and convergence into an axon tract or fascicle. These crucial first steps are required by axons of many developing brain nuclei, although little is known about how they are regulated. We identify two guidance signals critical for initial nucMLF axon guidance: laminin, a component of the extracellular matrix (ECM) through which axons grow, and transient axonal glycoprotein-1 (TAG-1), a cell adhesion molecule (CAM) present on axons.

The first axons to extend in the brain use the ECM and undifferentiated neuroepithelial cells as growth substrata and guidance sources. Laminins are heterotrimeric glycoproteins, consisting of α, β, and γ polypeptide chains, that are secreted into the ECM and form a major component of the ECM in the CNS [1]. *In vitro *studies have shown that laminin provides a permissive substratum for neuronal growth cones by binding and signaling through integrin receptors [2-6]. Moreover, laminin can modulate growth cone responsiveness to other extracellular guidance cues, such as netrins and ephrins [7,8]. Interestingly, contact with laminin stimulates axon formation in cultured hippocampal neurons [9], and netrin controls axon initiation in *Caenorhabditis elegans *[10], suggesting laminin or its modulation of netrin could contribute to the initial polarity of axon emergence. Mutations in particular laminin subunits in *C. elegans*, *Drosophila*, and zebrafish embryos [11-15], as well as antibody perturbation of laminin in grasshopper [16], cause axon guidance defects, indicating that laminins are important for axon guidance *in vivo*. However, our understanding of laminin function and its influence on growth cone behaviors *in vivo *is incomplete.

CAMs of the immunoglobulin (Ig) superfamily play crucial roles in mediating interaxonal relationships and communication between axons and their surrounding cells [17,18]. TAG-1 (or its chick homolog axonin-1) is a glycosyl-phosphatidylinositol (GPI)-linked IgCAM present on the surface of growing axons that promotes neurite outgrowth and interacts homophilically and heterophilically with a host of other cell surface molecules [19-21]. *In vitro *assays have shown that interactions with other IgCAMs, including L1 and NrCAM (NrCAM = neuron-glia related cell adhesion molecule), mediate axon outgrowth and cell-cell adhesion [22-26]. Furthermore, interactions of TAG-1 with L1 or NrCAM appear to be required *in vivo *for midline crossing by spinal commissural axons and for target selection by sensory neurons in the chick spinal cord [27-29]. Axon guidance defects in TAG-1 knockout mice have not been shown; however, neuronal migration in the caudal medulla is defective in these animals [30]. We have found previously that TAG-1 knockdown in zebrafish inhibits extension of sensory axons in the spinal cord, although whether this is mediated by a homophilic or heterophilic interaction is unknown [27-29,31]. Despite these studies demonstrating the importance of TAG-1, we have yet to fully understand whether or how TAG-1 controls directional axon guidance decisions or how it influences dynamic growth cone behaviors *in vivo*.

Here we investigate how TAG-1 and laminin-α1 influence growth cone behavior and axon outgrowth during the initial period of nucMLF axon development *in vivo*. Wild-type growth cone behaviors suggest that axon-axon interactions and possibly repulsion by surrounding tissue direct the initial pattern of nucMLF axon growth. Interestingly, TAG-1 knockdown causes defects in the directional extension and initial convergence of MLF axons that, in fixed tissue, appear identical to pathfinding errors previously observed in a laminin-α1 mutant, *bashful *[15]. However, *in vivo *time-lapse imaging of growth cone behaviors reveals that although there are some similar effects of TAG-1 or laminin-α1 loss of function, there are also distinct and significant differences. Our results suggest that TAG-1 functions to guide the direction of axon extension by interpreting environmental cues and also mediates positive axon-axon contacts. Laminin-α1 does not appear to affect axon-axon interactions, but does regulate neuronal polarity and influence growth cone responsiveness to surrounding cues.

## Results

### Characterization of normal MLF axon convergence

**Figure 1:**
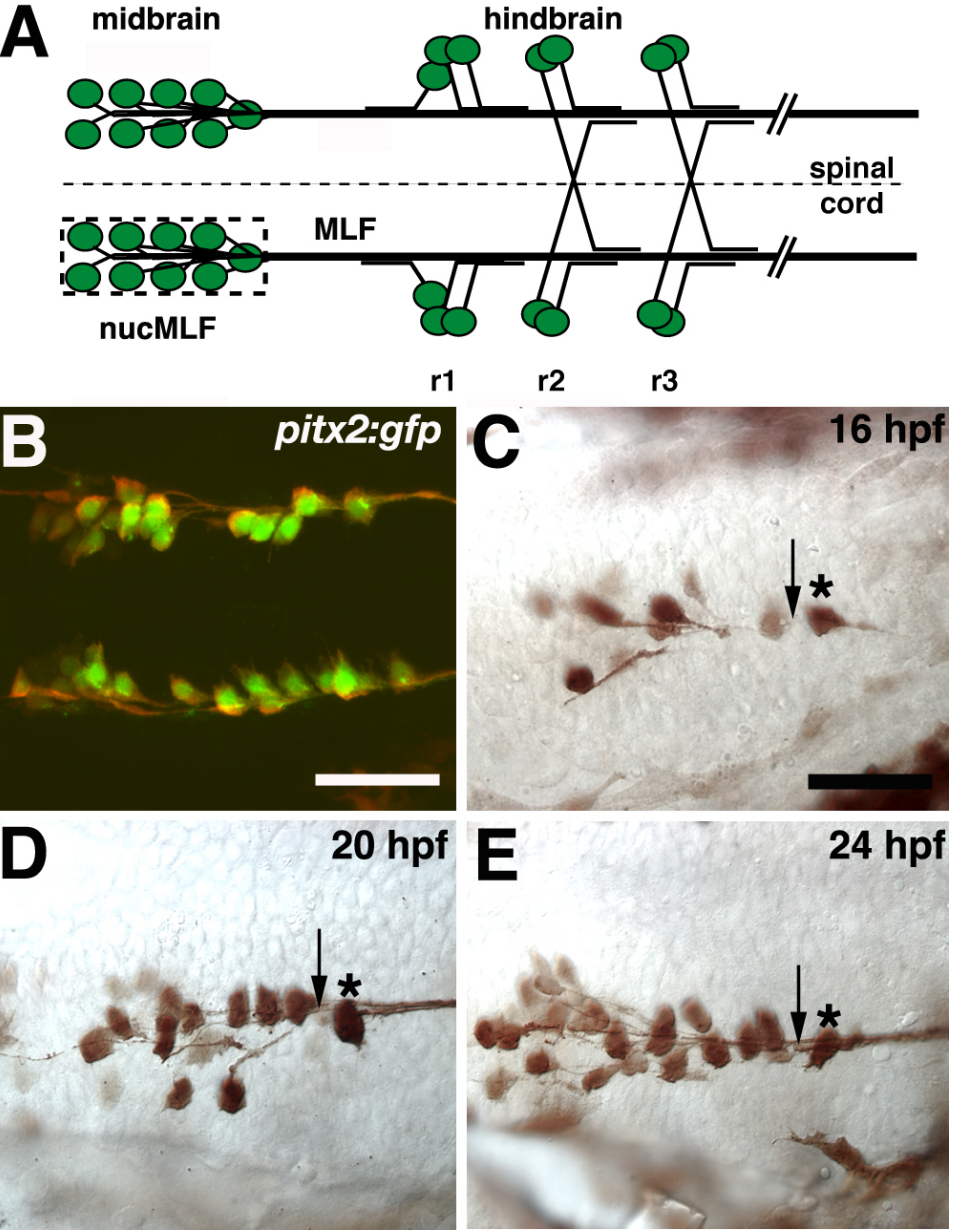
**Initial extension and convergence of MLF axons.(a-e) **Ventral views, anterior to the left. (a) Schematic representation of MLF axons and hindbrain axons that grow along the MLF. The dashed line denotes the ventral midline and the dashed box surrounds the 'nucMLF zone'. r = rhombomere. (b) Confocal projection of 20 hpf *Tg(pitx2c:gfp) *embryo stained with anti-GFP (green) and ZN-12 antibodies (red). (c-e) Whole mount preparations of *Tg(pitx2c:gfp) *embryos stained with anti-GFP at 16 (c), 20 (d), and 24 (e) hpf. Midline is up. Asterisks denote the caudal-most nucMLF cell and arrows indicate the 'convergence point'. Scale bar = 25 μm.

To investigate the guidance cues and growth cone behaviors involved in the initial outgrowth of axons from an early developing brain nucleus, we studied the formation of the zebrafish MLF, one of the first axon tracts to develop in the zebrafish central nervous system (CNS; Figure 1a) [32]. The MLF arises from bilateral clusters of neuronal cell bodies, called the nucMLF, that lie in the ventral midbrain. At the onset of MLF formation (16 hours post fertilization (hpf)), the nucMLF consists of 6–8 neurons and by 24 hpf the cluster has grown to approximately 30–35 neurons [33,34]. During the initial wave of axonogenesis (16–30 hpf), unipolar nucMLF cells each extend an axon caudally along the ipsilateral ventral neural tube. The caudal-most nucMLF cell extends the leading MLF axon into the hindbrain and then the more rostrally positioned cells extend axons that fasciculate with the leading axon [35]. We have previously reported that Semaphorin3D, which is expressed both rostral and medial to the nucMLFs, plays a role in repelling nucMLF axons in the caudal direction [36,37]; however, nothing is known about other extracellular cues involved in guiding the initial pathfinding decisions of nucMLF axons. Here, we further investigate guidance mechanisms that control how nucMLF axons initially extend in the correct direction and fasciculate with the leading nucMLF axon.

We first characterized the pattern of nucMLF axon outgrowth by examining fixed embryos at stages between 16 and 24 hpf. To specifically visualize nucMLF neurons, we used a transgenic line in which a *pitx2 *promoter construct drives green fluorescent protein (GFP) expression (*Tg(pitx2c:gfp)*) in nucMLF neurons (Figure 1b–e; see Materials and methods). We verified that all nucMLF neurons are labeled by double labeling embryos with antibodies against ZN-12, a marker of nucMLF neurons, and GFP (Figure 1b). At the onset of nucMLF axonogenesis, cell bodies each extended one axon in the direction of the caudal-most nucMLF cell body (Figure 1c–e, asterisk), which extended its own axon into the hindbrain, parallel to the ventral midline. In their initial trajectory, the earliest nucMLF axons typically did not contact neighboring axons since their cell bodies were positioned at some distance from one another, but later as these axons approached the caudal-most nucMLF cell and as the number of nucMLF cells increased, axon-axon contact was prevalent. Interestingly, the axons did not appear to extend into the tissue surrounding the nucMLF. Rather, they appeared to extend directly toward the caudal-most nucMLF cell body and eventually converged on a point just rostral to this cell (Figure 1c–e, arrow). In our subsequent analysis, we will refer to this point as the convergence point. Beyond the convergence point, MLF axons extend into the hindbrain as a tight fascicle.

### Axon-axon interactions and the surrounding tissue contribute to the convergence of nucMLF axons

**Figure 2:**
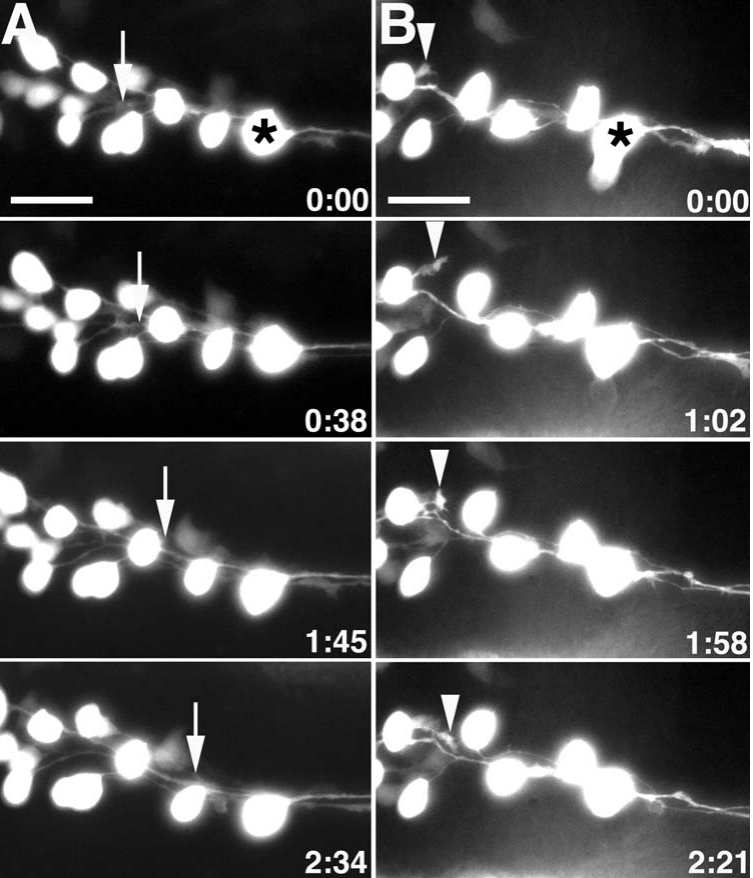
**MLF growth cones fasciculate along neighboring MLF axons and are inhibited by surrounding tissue.(a, b) **Images from time-lapse sequence of MLF axon outgrowth in uninjected *Tg(pitx2c:gfp) *embryos. Ventral views, anterior to the left, midline is up. Asterisks denote the caudal-most nucMLF cell. (a) The arrows indicate the position of the growth cone that fasciculates along a neighboring MLF axon. (b) The arrowheads label the growth cone that is repelled by surrounding tissue. The time stamp shows hours: minutes. Scale bar = 25 μm.

To better understand nucMLF growth cone behaviors and gain insight into potential guidance factors that might control initial axon direction, convergence, and fasciculation, we performed *in vivo *time-lapse imaging of these neurons. We reasoned that specific growth cone behaviors (that is, retraction, pausing, branching, and so on) would reflect underlying molecular mechanisms responsible for these behaviors. To visualize growing nucMLF axons *in vivo*, we removed the yolk cell from *Tg(pitx2c:gfp) *embryos and imaged the nucMLF from the ventral side of the embryo proper (see Materials and methods for details), beginning at approximately 16 hpf. Consistent with the fixed staging series, each nucMLF neuron extended one axon from its caudal side, which grew towards the convergence point. Once growth cones contacted neighboring MLF axons, they maintained these contacts and extended along the axon *en route *to the convergence point. This behavior was highly conserved and these interactions appeared to expedite growth towards the convergence point, suggesting that axon-axon contacts may be important for MLF formation (n = 11 embryos, 32 growth cones; Figure 2a and Additional file 1). In some cases, axons extended from cell bodies that were positioned up to 25 μm from their nearest neighbor, and, therefore, the majority of their path towards the convergence point was not along a neighboring axon. This observation suggested that axon-axon interactions were not the only factor required for proper convergence. Indeed, we found that if a wild-type growth cone extended aberrantly into the surrounding tissue, away from the convergence point, it was able to retract and redirect its growth in the appropriate direction without contact with other axons (Figure 2b and Additional file 2). This result suggests that nucMLF axons are guided by surrounding tissues, perhaps by repulsive cues that funnel them towards the convergence point.

### TAG-1 and laminin-α1 are required for the initial convergence of nucMLF axons

**Figure 3:**
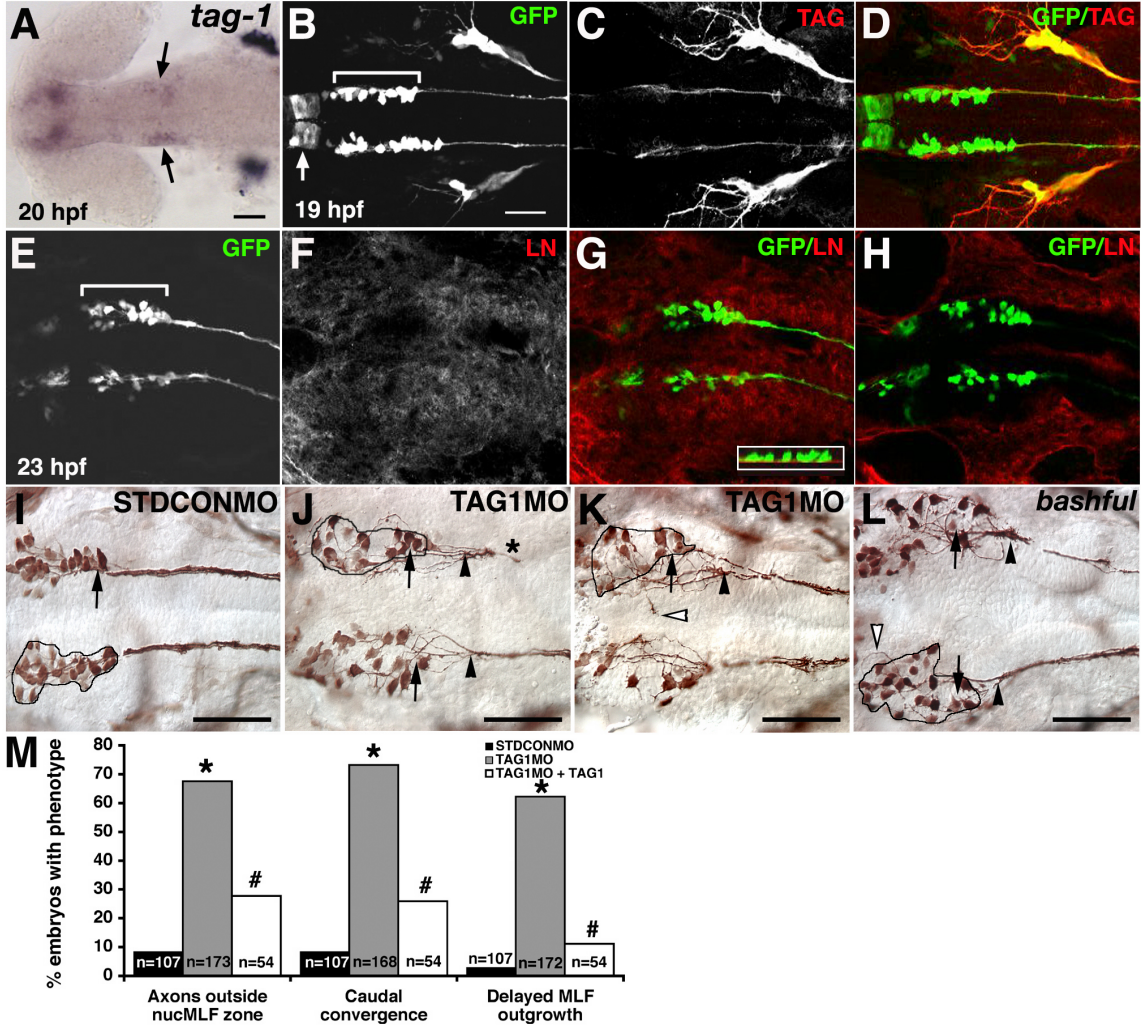
**TAG-1 or laminin-α1 loss of function disrupts normal MLF axon convergence.(a-l) **Ventral views, anterior to the left. (a) *In situ *hybridization for *tag-1 *in 20 hpf embryo. Arrows indicate expression in nucMLF. (b-d) Confocal projections of 19 hpf *Tg(pitx2c:gfp) *embryos labeled with anti-GFP (green) and anti-TAG-1 (red) antibodies. The arrow indicates unidentified cells in the diencephalon expressing the *pitx2c:gfp *transgene. The brackets indicate the extent of nucMLF. (e-h) Single confocal plane of nucMLF region in 23 hpf *Tg(pitx2c:gfp) *embryos labeled with anti-GFP (green) and anti-laminin (red). (e-g) The focal plane is at the ventral surface of the neuroepithelium. The inset in (g) is a 90 degree rotation of a z-reconstruction, with ventral down, showing the laminin underlying the nucMLF. (h) The focal plane is 4 μm dorsal to the plane in (e-g), and shows that laminin is not concentrated within the neuroepithelium. (i-l) Whole mount preparations of STDCONMO injected embryos (i), TAG1MO injected embryos (j, k), and *bal;Tg(pitx2c:gfp) *embryos (l) labeled with an anti-GFP antibody. The outline defines the 'nucMLF zone'. Arrows indicate the normal convergence point. Filled arrowheads denote the actual convergence point. Open arrowheads label examples of MLF axons outside of the nucMLF zone. The asterisk indicates stunted MLF extension. (m) Quantification of the average percentage of embryos with MLF phenotypes. *P < 0.001, two sample binomial comparison versus STDCON. ^#^*P *< 0.001, two sample binomial comparison versus TAG1MO. N equals the number of embryos. Scale bars = 50 μm. The scale bar in (b) is for (b-h).

Given the significant axon-axon interactions we observed in the live imaging experiments, we asked whether TAG-1, a GPI-linked Ig superfamily CAM known to mediate axon-axon interactions [19], was critical for the initial growth and convergence of MLF axons. *tag-1 *mRNA is expressed by a subset of neurons in the zebrafish brain, including the nucMLF (Figure 3a) [38]. We examined the expression of TAG-1 protein during initial stages of MLF development (16–24 hpf), and found that it is expressed on the nucMLF axons and weakly on the nucMLF cell bodies throughout these stages (Figure 3b–d, and data not shown). In addition, TAG-1 is expressed on axons from the nucleus of the tract of the postoptic commissure (nucTPOC) that begin to extend past the lateral side of the nucMLF around 19–20 hpf, and on a couple of cells lateral to the nucMLF, but not otherwise in the surrounding neuroepithelium. We injected *Tg(pitx2c:gfp) *embryos at the 1–4 cell stage with a translation blocking TAG-1 morpholino antisense oligonucleotide (TAG1MO) [31], allowed them to develop to 24 hpf, and examined the pattern of nucMLF axon outgrowth in fixed embryos by immunolabeling with an anti-GFP antibody. We have previously shown that injection of TAG1MO, but not a standard control morpholino (STDCONMO), eliminates TAG-1 immunolabeling, indicating the loss of TAG-1 protein [31]. TAG-1 knockdown caused several defects in nucMLF axon initial growth and convergence, but did not appear to alter the number of nucMLF neurons (Figure 3i–k, m). Normally, nucMLF axons initially extend only within the area containing the nucMLF cell bodies (called the 'nucMLF zone'; outlined in Figure 3i–l) prior to reaching the convergence point. However, after TAG-1 knockdown, nucMLF axons aberrantly extended into the tissue medial, lateral, and rostral to the nucMLF zone (Figure 3k, open arrowhead). This phenotype was observed in 67.6% (n = 173) of TAG1MO injected embryos, compared with only 8.4% (n = 107) of STDCONMO injected embryos (Figure 3i–k, m). In addition, the axons converged at a point more caudal than the typical convergence point in 73.2% (n = 168) of embryos injected with TAG1MO, while injection of STDCONMO caused this defect in only 8.4% (n = 107) of embryos (Figure 3i–k, arrowheads, and Figure 3m).

Knockdown of TAG-1 also significantly stunted nucMLF axon outgrowth. Normally, the leading edge of the MLF has reached the anterior spinal cord by 24 hpf [34]; however after TAG-1 knockdown, MLF axons had not extended beyond the anterior hindbrain in 62.2% (n = 172) of embryos, compared with only 2.8% (n = 107) of STDCONMO injected embryos (Figure 3j, m). To determine whether these defects were specifically due to TAG-1 knockdown and not to nonspecific morpholino effects, we attempted to rescue the defects by co-injecting TAG1MO with a TAG-1 mRNA construct that did not include the majority of the TAG1MO binding region. TAG-1 overexpression did not cause any non-specific gross morphological changes, but did significantly reduce the percentage of embryos with nucMLF defects, suggesting that these defects are specific to TAG-1 knockdown (Figure 3m). Furthermore, knockdown of L1, another Ig superfamily CAM that is expressed by the nucMLF [39], did not cause the above defects, although it did cause MLF defasciculation in the hindbrain [35], suggesting that not all CAMs expressed by these neurons are required for MLF axon convergence. Together, these data suggest that TAG-1 is required for the efficient convergence of nucMLF axons and that it potentially functions to promote positive axon-axon interactions and/or allow nucMLF axons to interpret signals from the surrounding tissue.

Interestingly, the nucMLF axon defects induced by TAG-1 knockdown appeared very similar to defects we previously observed in *bashful *(*bal*) embryos, which have a mutation in the laminin-α1 subunit (Figure 3l) [15,40]. Laminin is expressed broadly in the basal lamina along the ventral surface of the neuroepithelium, directly apposed to the nucMLF and the MLF axons during all stages examined (16–24 hpf; Figure 3e–h, and data not shown). In *bal *mutants, the nucMLF axons also appear to extend aberrantly outside the nucMLF zone before eventually converging in the anterior hindbrain. Moreover, these axons appear defasciculated and stalled in the hindbrain. The similar defects suggest that TAG-1 and laminin-α1 may function together to guide the initial convergence of nucMLF axons or that they may have similar influences on growth cone behavior to produce comparable pathfinding defects. However, the fixed tissue analysis of nucMLF axon defects was insufficient to address these questions.

### NucMLF growth cones exhibit behavioral defects after TAG-1 or laminin-α1 loss of function

**Figure 4:**
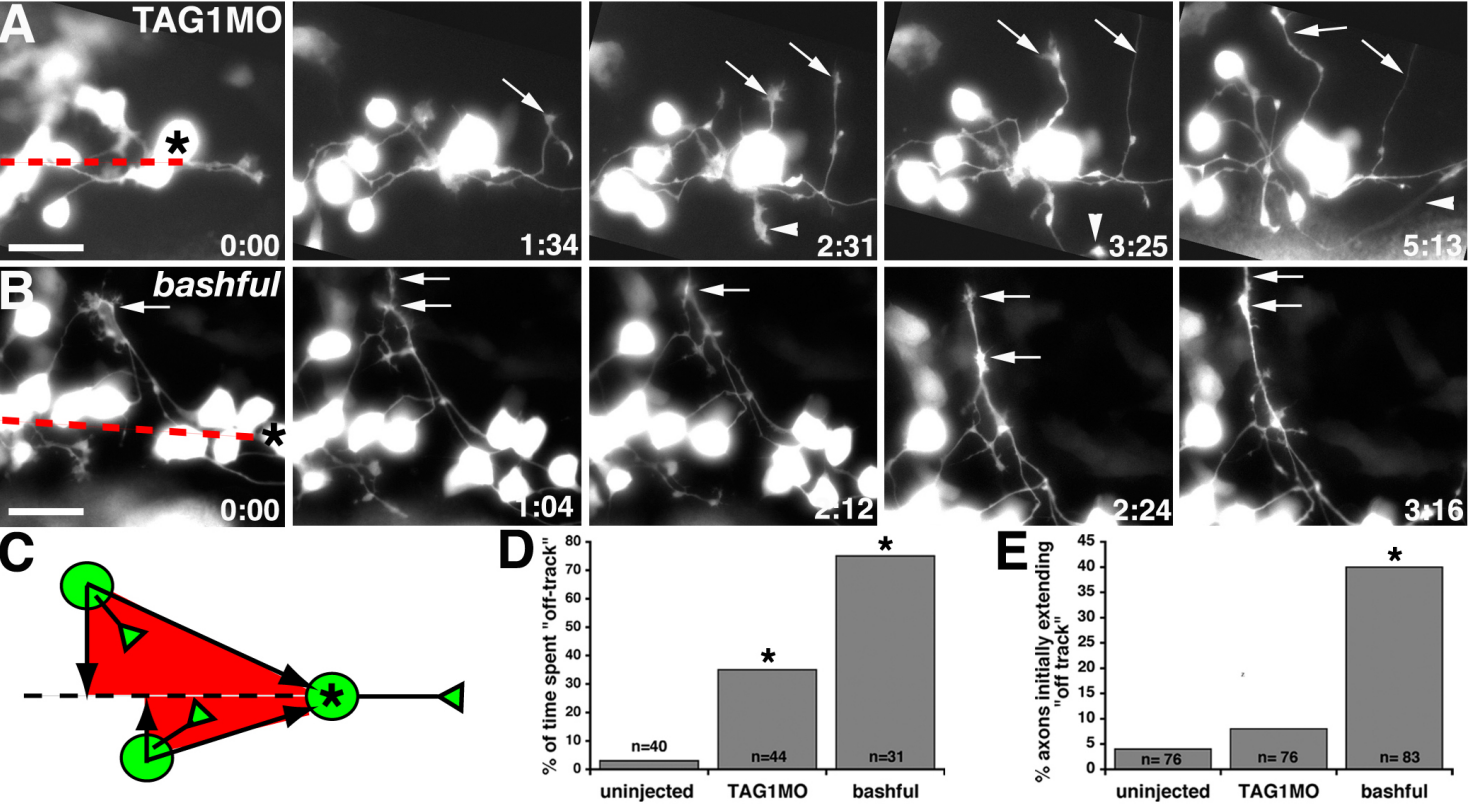
**TAG-1 or laminin-α1 loss of function causes misdirected MLF axon outgrowth.(a, b) **Images from timelapse sequence of MLF axon outgrowth in TAG1MO injected (a) and *bal;Tg(pitx2c:gfp) *(b) embryos. Ventral views, anterior to the left, midline is up. Asterisks denote the caudal-most nucMLF cell. The dashed red line marks the nucMLF centerline. Arrows indicate MLF axons that wander into surrounding tissue and do not converge within timelapse duration. Arrowheads label an MLF axon that extends into lateral tissue, but eventually converges. The time stamp shows hours: minutes. **(c) **Schematic representation defining on-track (red shaded region) versus off-track regions. **(d, e) **Quantification of the average percentage of time MLF axons grew off-track (d) and the average percentage of axons that initially emerged from their cell body in an off-track position (e). *P < 0.001, two sample binomial comparison versus uninjected. N equals the number of MLF axons. Scale bar = 25 μm.

To determine how TAG-1 and laminin-α1 loss of function influences nucMLF growth cone behaviors, we performed *in vivo *time-lapse imaging in TAG1MO injected *Tg(pitx2c:gfp) *embryos and *bal*;*Tg(pitx2c:gfp) *embryos. Interestingly, live imaging results indicate these cues play both overlapping and unique roles in regulating nucMLF growth cone behaviors to cause the apparently identical defects observed in fixed tissue. In TAG1MO injected or *bal *embryos, axons extended in aberrant directions and into the tissue surrounding the nucMLF zone, without retracting or immediately correcting their growth direction (Figure 4a, b and Additional files 3, 4). Some of these misdirected axons eventually converged with other nucMLF axons that had extended properly (Figure 4a, arrowhead), while others approached or crossed the ventral midline (Figure 4a, b, arrows) or grew laterally after exiting the nucMLF zone. Interestingly, axons extended into medial (30%, n = 49 axons in 10 embryos) and lateral (23%, n = 49 axons in 10 embryos) tissues with comparable frequency after TAG-1 knockdown. However, in *bal *embryos, the majority of axons extending into surrounding tissue grew towards the ventral midline (53%, n = 80 axons in 8 embryos; 16% extended laterally), and in some cases, these axons accelerated as they neared the midline. To quantify the directional outgrowth errors, we determined the average percentage of time that axons extended in the correct direction ('on-track') and whether each MLF axon initially emerged from its cell body in an 'on-track' or 'off-track' position. We defined 'on-track' as growth within a region defined by a line extending from the cell body at 90 degrees to the nucMLF centerline and another line connecting the cell body to the convergence point (Figure 4c). The nucMLF centerline was defined as an imaginary line drawn along the MLF and extending rostrally through the nucMLF (Figure 4a, c, dashed line). Growth outside of this region or across the nucMLF centerline was considered 'off-track'. On average, axons spent significantly more time growing off-track after TAG-1 or laminin-α1 loss of function (Figure 4d). Axons from uninjected embryos extended off-track only 3% of the time (n = 40 axons in 11 embryos). However, axons in TAG1MO injected and *bal *embryos grew off-track 35% (n = 44 axons in 10 embryos) and 75% (n = 31 axons in 8 embryos) of the time, respectively. In *bal *embryos, a significant number of axons (40%, n = 83 axons in 8 embryos; Figure 4e) initially emerged from their cell bodies in an incorrect direction, which may, at least in part, explain why these axons grew off-track the majority of the time measured. In contrast, the majority of axons in TAG1MO injected embryos emerged from their cell bodies in an on-track position and later extended off-track. Taken together, these data suggested that TAG-1 and laminin-α1 may both be required for nucMLF growth cones to be guided by the surrounding tissue and that laminin-α1 is important for establishing the initial polarity of nucMLF neurons.

### TAG-1, but not laminin-α1, affects nucMLF axon-axon interactions

**Figure 5:**
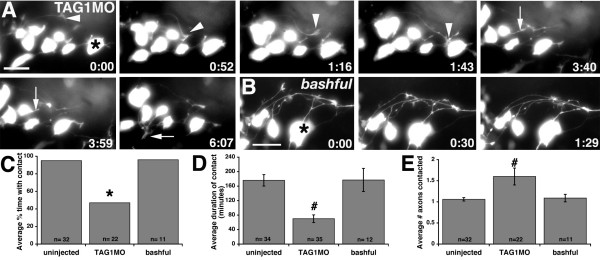
**TAG-1 knockdown causes defects in MLF axon-axon interactions.(a, b) **Images from time-lapse sequence of MLF axon outgrowth in TAG1MO injected (a) and *bal;Tg(pitx2c:gfp) *(b) embryos. Ventral views, anterior to the left, midline is up. Asterisks denote the caudal-most nucMLF cell. Arrowheads label a growth cone that repeatedly samples neighboring axons, but fails to maintain these interactions. Arrows label a growth cone that contacts another MLF axon, subsequently retracts, and then emerges from the opposite side of the neuron. The time stamp shows hours: minutes. **(c-e) **Quantification of the average percentage of time each growth cone contacted another MLF axon after contact was initiated (c), the average duration of these contacts (d), and the average number of axons each growth cone contacted (e). *P < 0.001, two sample binomial comparison versus uninjected. ^#^*P *< 0.001, two-tailed *t*-test versus uninjected. Error bars represent the standard error of the mean. N equals the number of axons (c, e) and growth cone-axon contacts (d). Scale bar = 25 μm.

The live imaging experiments also revealed a role for TAG-1, but not laminin-α1, in regulating axon-axon interactions among converging nucMLF axons. In TAG1MO injected embryos, we commonly observed growth cones contacting neighboring axons and then subsequently retracting (Figure 5a and Additional file 5). In many cases, growth cones would sample multiple other axons, but fail to maintain any of the interactions. None of these behaviors were observed in *bal *embryos where aberrantly guided axons maintained their interactions once they contacted other axons (Figures 4b and 5b, and Additional files 4 and 6). To quantify the maintenance of axon-axon interactions, we calculated the average percentage of time in which growth cones were in contact with neighboring axons once contact was initiated, the average duration of each contact, and the average number of axons contacted per growth cone. On average, axons in TAG1MO injected embryos were in contact with neighboring axons only 47% (n = 22 axons in 10 embryos) of the time compared with 95% (n = 32 axons in 11 embryos) and 96% (n = 11 axons in 8 embryos) of the time in uninjected and *bal *embryos, respectively (compare Figures 5c and 2a). Moreover, the average duration of these contacts was 70 minutes (n = 35 axon contacts in 10 embryos) in TAG1MO injected embryos versus 176 minutes (n = 34 axon contacts in 11 embryos) and 177 minutes (n = 12 axon contacts in 8 embryos) in uninjected and *bal *embryos, respectively (Figure 5d). Finally, prior to converging, each growth cone contacted an average of 1.06 axons (n = 32 growth cones in 11 embryos) in uninjected and 1.09 axons (n = 11 growth cones in 8 embryos) in *bal *embryos (Figure 5e). However, after TAG-1 knockdown, each growth cone sampled 1.6 axons (n = 22 growth cones in 10 embryos; Figure 5e), suggesting that growth cones repeatedly sample neighboring axons despite their inability to maintain these interactions. The failed axon interactions in TAG1MO injected embryos may contribute to their increased off-track extension since these axons often changed their trajectory and extended away from the nucMLF centerline after breaking contact with a neighboring axon. Moreover, these axon-axon defects may have potential additional consequences in terms of neuronal polarity. In one case, we observed the growth cone of a newly emerged MLF axon contact a neighboring axon and then collapse (Figure 5a, arrow). After collapsing, the axon retracted to its cell body and then what appeared to be a new axon emerged from the opposite side of the cell body, consequently changing the neuron's polarity. We never observed such polarity changes in uninjected embryos, nor did we ever observe a nucMLF neuron extend multiple axons and then prune all but one axon in any of the groups. Collectively, these observations suggested that TAG-1 knockdown reduced positive axon-axon interactions, which can cause nucMLF axons to grow off-track and possibly influence nucMLF neuronal polarity.

### nucMLF axon branching increases in *bal*, but not after TAG-1 knockdown

**Figure 6:**
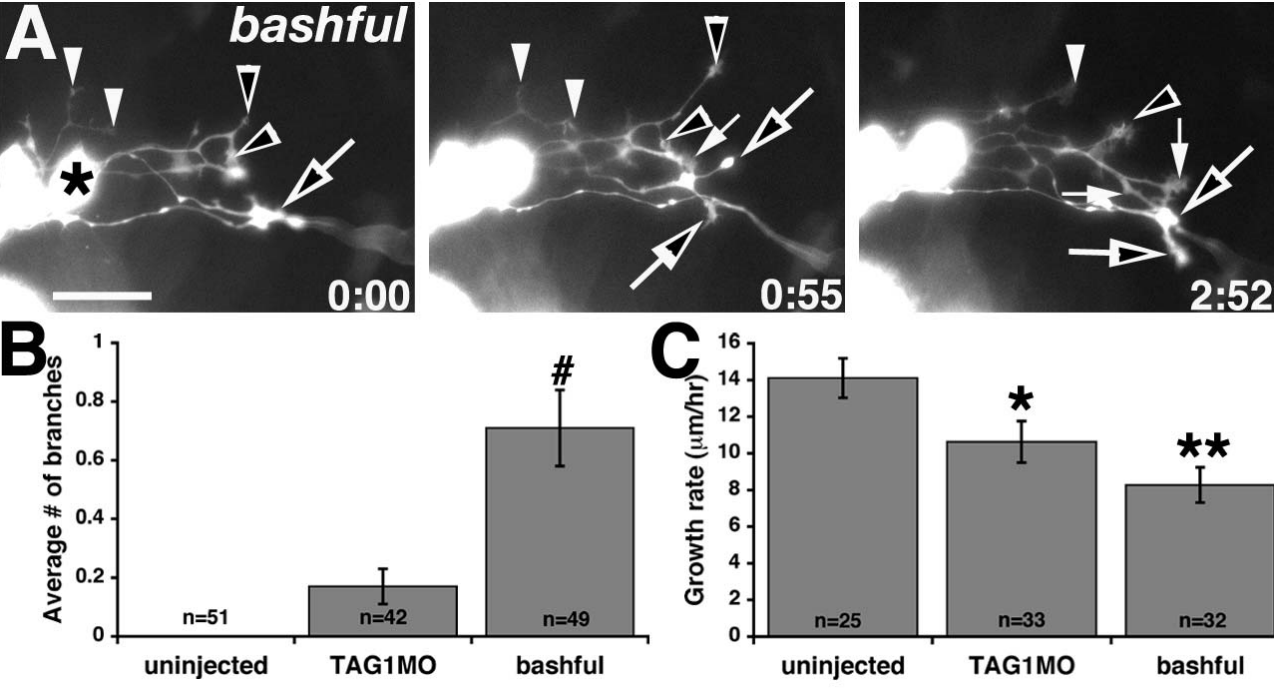
***bal *****embryos have excessively branched axons and MLF growth rates are reduced by TAG-1 or laminin-α1 loss of function.(a) **Images from timelapse sequence of MLF axon outgrowth in a *bal;Tg(pitx2c:gfp) *embryo. Ventral views, anterior to the left, midline is up. The asterisk denotes the caudal-most nucMLF cell. Open and filled arrows and arrowheads label branches from individual axons. The time stamp shows hours: minutes. **(b, c) **Quantification of the average number of branches per axon (b) and the average growth rate of MLF axons (c). ^#^*P *< 0.001, Kruskal-Wallis ANOVA versus uninjected. *P < 0.05, **P < 0.001, two-tailed *t*-test versus uninjected. Error bars represent the standard error of the mean. N equals the number of axons. Scale bar = 25 μm.

Typically, nucMLF axons do not branch as they initially converge in the midbrain and extend through the hindbrain. Only much later in development do they normally develop collateral branches that innervate the spinal cord [41]. However, in *bal*, we commonly observed axons that extended collateral branches or growth cones that split and produced divergent axons (Figure 6a and Additional file 7). nucMLF axons did not branch significantly in uninjected or TAG1MO injected embryos. Branches were counted if they lasted for at least ten minutes and many of the branches lasted for the duration of the imaging session (up to six hours). On average, axons in *bal *embryos each extended 0.71 branches (n = 49 axons in 8 embryos), compared to 0 branches in uninjected embryos (n = 51 axons in 11 embryos) and 0.17 branches in TAG1MO injected embryos (n = 42 axons in 10 embryos) (Figure 6b). In *bal *embryos, approximately 54% (n = 49 axons in 8 embryos) of axons extended at least one branch, and 19% had more than one branch. The majority of these branches extended from the axon shaft. These observations suggest that the presence of laminin-α1 normally suppresses axon branching, and that TAG-1 does not influence axon branching during nucMLF axon convergence.

### nucMLF axons have reduced growth rates in TAG-1 knockdown and *bal *embryos

The stunted MLF outgrowth phenotype we observed in the fixed tissue analysis of TAG1MO injected and *bal *embryos (Figure 3i, j, m) [15] may be due, in part, to their initial extension in incorrect directions, but a reduced growth rate may also contribute to this phenotype. Therefore, we quantified the growth rate of axons in uninjected, TAG1MO injected, and *bal *embryos from their initial extension until they reached the convergence point or an equivalent position in the rostral-caudal axis (Figure 6c). Axons in uninjected embryos grew at an average of 14.11 ± 1.08 μm/h (Figure 6c). However, axons in TAG1MO injected or *bal *embryos grew significantly slower at an average of 10.63 ± 1.13 μm/h and 8.27 ± 0.96 μm/h, respectively (Figure 6c). Thus, in addition to their inefficient pathfinding towards the convergence point, reduced growth rates likely contribute to the retarded MLF axons observed after TAG-1 or laminin-α1 loss of function.

## Discussion

In this study, we use *in vivo *time-lapse imaging to show how TAG-1 and laminin-α1 affect dynamic growth cone behaviors, initial axon direction, and interactions among nucMLF axons in the zebrafish brain. Normally, nucMLF axons converge and fasciculate soon after extending from their cell bodies. Our results suggest this growth pattern is driven by a combination of guidance by surrounding tissue and axon-axon fasciculation. TAG-1 or laminin-α1 loss of function disrupted this stereotypical outgrowth pattern. First, TAG-1 or laminin-α1 loss of function caused MLF axons to aberrantly extend into the surrounding tissue. TAG-1 knockdown also caused MLF growth cones to retract from neighboring MLF axons, which led to aberrant directional extension of MLF axons and possibly influenced changes in neuronal polarity. Axon-axon interactions appeared normal in *bal *embryos; however, neuronal polarity was affected, since MLF axons often emerged from their cell bodies in the incorrect direction. MLF axons were also excessively branched in *bal *embryos. Finally, MLF axons in TAG1MO injected or *bal *embryos exhibited reduced growth rates and were stalled in the hindbrain. Taken together, these results reveal critical roles for TAG-1 and laminin-α1 in controlling specific growth cone behaviors, neuronal polarity, and directional outgrowth to guide proper formation of a major CNS pathway.

Interestingly, TAG-1 may serve dual functions in mediating nucMLF axon pathfinding as it appears to be required for sensing a signal in the surrounding tissue and for mediating axon-axon interactions. Several Ig superfamily CAMs, including TAG-1, have been shown to mediate axon fasciculation via homophilic and heterophilic binding partners [17-19]. Therefore, the defective nucMLF axon-axon interactions caused by TAG-1 knockdown were not surprising. It is highly possible that TAG-1 promotes adhesion among nucMLF axons either homophilically or heterophilically, although a heterophilic partner has not been determined in this case. The apparent consequences of these failed interactions were more intriguing. Often, growth cone retraction upon contacting a neighboring axon was followed by a change in direction and extension into the surrounding tissue, suggesting that these defective interactions may have contributed to further pathfinding errors. Furthermore, in a few cases, defective axon-axon interactions led to the complete retraction of the original axon and the emergence of a new axon on the opposite side of the neuron, thus altering the polarity and directional extension of nucMLF axons. Collectively, these results exemplify the significance of TAG-1 mediated axon-axon interactions not only to fasciculation, but also to directional guidance and neuronal polarity. Finally, it is possible that TAG-1 mediates interactions between nucMLF and nucTPOC axons, which also extend through the midbrain and join the MLF. However, nucTPOC axons grow into this region 2–3 hours after the initial outgrowth of nucMLF axons and appeared unaffected by TAG-1 knockdown.

In addition to this expected role for TAG-1, our results also suggest TAG-1 is required for nucMLF axons to sense a guidance signal from the environment because TAG-1 knockdown also caused nucMLF axons to grow in aberrant directions without first contacting other axons. This signal potentially could be an inhibitory cue located in tissue surrounding the nucMLF and/or an attractive cue emanating from the convergence point. This finding is interesting because TAG-1 is not known to function as a receptor for repulsive or attractive ligands. However, related Ig superfamily CAMs, such as L1 and NrCAM, have recently been shown to be required receptor components for repulsive semaphorin signaling [42-45]. Thus, these observations raise the intriguing possibility that TAG-1 may also serve as a receptor component for repulsive and/or attractive ligands *in vivo*.

Laminin also appears to be involved in preventing MLF axon growth into inappropriate surrounding tissues and orienting outgrowth direction. Laminin is expressed throughout the basal lamina upon which MLF axons extend, and thus it could function directly to guide nucMLF axons by signaling via integrins or other receptor molecules on nucMLF growth cones. Alternatively, laminin-α1 may affect axons indirectly through more global effects on brain organization or by binding and presenting other guidance signals. An interesting possibility is that laminin-α1 may regulate growth cone responsiveness to other cues expressed by the surrounding tissue, such as netrin or ephrin, which have been shown to elicit repulsion in the presence of laminin [7,8]. Without functional laminin-α1, nucMLF axons may be attracted or non-responsive to such cues. Interestingly, in *bal*, misguided axons appeared biased to extend towards the ventral midline rather than wandering in all directions equally. This observation suggests that laminin-α1 indirectly regulates axon repulsion by another cue in the midline region because laminin-α1 is expressed broadly, not only at the midline [46].

Loss of laminin-α1 also caused defects in polarity and branching. Establishing appropriate neuronal polarity is arguably the first step towards properly guiding an axon because it determines the initial direction of axon outgrowth. Normally, almost all nucMLF neurons immediately extended their axon in the direction of the convergence point. However, in *bal*, we observed a significant number of neurons that initially extended axons in aberrant directions, suggesting that laminin-α1 is critical for establishing the proper polarity of these neurons. Again, whether nucMLF neuronal polarity is directly or indirectly regulated by laminin-α1 remains to be determined. Recently, netrin was shown to regulate neuronal asymmetry and define the site of axon formation [10]. It is possible that these roles may also require the presence of laminin, similar to netrin-mediated repulsion. Functionally, the nucMLF polarity defect may also have contributed to the increased percentage of time that axons extended off-track. In addition to polarity defects, axons were excessively branched in *bal*. It might be surprising that loss of laminin-α1, which usually functions as a permissive growth substratum, would directly stimulate increased branching. However, it is possible that the loss of laminin-α1 created an inhibitory substratum causing growth cone stalling or retraction, which has been correlated with back branching [47,48]. These observations reveal novel roles for laminin-α1 in establishing neuronal polarity and regulating axon branching.

TAG-1 or laminin-α1 loss of function ultimately caused nucMLF axons to prematurely stall in the hindbrain. Interestingly, the stalled axons were almost always observed in the anterior hindbrain, instead of being positioned along a continuum from the anterior hindbrain to the anterior spinal cord, where they should have reached by 24 hpf. This observation suggests that an additional cue may be required to promote axon extension through this region and that late arriving axons may miss the temporal expression window of this factor. Alternatively, nucMLF axons may become non-responsive to the potential factor in the absence of TAG-1 and/or laminin-α1 function. In any case, it is interesting that the position of stalled nucMLF axons is the same in embryos deficient for TAG-1 or laminin-α1.

## Conclusion

Collectively, our live imaging data reveal that TAG-1 and laminin-α1 loss of function cause some similar defects in nucMLF growth cone behavior, while also causing defects unique to the loss of each gene. The fact that both contributed to restricting nucMLF axon outgrowth to within the nucMLF zone might suggest that TAG-1 and laminin-α1 may function cooperatively to mediate inhibition of growth cones by surrounding tissue. One possibility is that TAG-1 and laminin-α1 act in separate signaling pathways, with common and distinct downstream effectors. Alternatively, laminin-α1 in the environment may directly bind TAG-1 on nucMLF growth cones, although no such interaction has been demonstrated. Instead, laminin-α1 may indirectly regulate the presentation of a binding partner of TAG-1. Future experiments will be required to address these alternative mechanisms.

## Materials and Methods

### Animals

Zebrafish (*Danio rerio*) were maintained in a laboratory breeding colony on a 14/10 h light/dark cycle. Embryos were maintained at 28.5°C and staged as described previously [49]. *bashful *mutant embryos were obtained from fish carrying the *bal*^uw-1 ^allele [15], which contains a mutation that results in a predicted protein truncation at amino acid 1,424 of laminin-α1 [50]. Animals were handled in accordance with guidelines set forth by NIH and IACUC, and the animal use protocol was approved by the University of Wisconsin Animal Care and Use Committee (assurance number A3368-01).

### Generation of Tg(*pitx2c:gfp*) transgenics

The *Tg(pitx2c:gfp) *transgenic line was generated using an internal promoter of the *pitx2 *gene that specifically produces the *pitx2c *isoform [51]. This promoter was obtained by amplifying a region from exon 2 to exon 4 of the *pitx2 *gene from a PAC clone. This fragment was fused to the enhanced GFP (EGFP) coding sequence followed by the SV40p(A) signal sequence from pEGFP-N1 (Clontech, Mountain View, CA, USA) and cloned into pBSIIKS- (Stratagene, La Jolla, CA, USA). The primers used for PCR amplification of the pitx2c promoter were: 5'-AGGGTACCGGACTCCCACTGCCGCAAACT-3' and 5'-TCTAGAGCTCTGTAATGCAAAAGGAAACAC-3'. The primers used to amplify the EGFP-SV40p(A) fragment were: 5'-TAACGAGCTCATGGTGAGCAAGGGCGAGGA and 5'-CAGAATTCTGAGTTTGGACAAACCACAAC-3'. To generate transgenic zebrafish, the vector sequence was removed from the *pitx2c:egfp *construct prior to injection into embryos at the one-cell stage. Three independent lines were isolated that all showed expression of EGFP in the trigeminal ganglia and the nucMLF.

### Immunohistochemistry

For whole-mount immunohistochemistry, embryos were fixed in 4% paraformaldehyde overnight, blocked in 5% sheep serum and 2 mg/ml bovine serum albumin in phosphate buffered saline with 0.1% Tween, and incubated overnight (4°C) in anti-GFP (1:1,000; Sigma, St Louis, MO, USA), ZN-12 (1:250; Zebrafish International Zebrafish Center, Eugene, OR, USA) or anti-TAG1 (1:500; [52]). Antibody labeling was performed with the Vectastain IgG ABC immunoperoxidase labeling kit (Vector Laboratories, Burlingame, CA, USA). For fluorescent double labeling, Alexa-conjugated secondary antibodies (4 μg/ml; Invitrogen-Molecular Probes, Carlsbad, CA, USA) were used to bind primary antibodies.

### Morpholino antisense

Morpholino oligonucleotides were synthesized by Gene Tools (Corvallis, OR, USA). To knockdown TAG-1, we used a morpholino sequence (TAG1MO; 5'-CCACACCCA-GACCAGACACTTATTT-3') that has been previously reported and shown to block TAG-1 protein [31]. As a control, we injected a standard control morpholino (STDCONMO; 5'-CCTCTTACCTCAGTTACAATTTATA-3'). Morpholino oligos were injected into newly fertilized embryos at the one- to four-cell stage as described previously [53]. Optimal concentrations for the TAG1MO were determined by titrating the dose from 1–10 ng per embryo; 10 ng injections appeared to cause non-specific toxicity, 2.5–5 ng injections appeared to cause robust defects without non-specific cell death, and 1 ng injections caused partial, but insignificant, defects. Therefore, we injected TAG1MO at approximately 2.5–5 ng to elicit full knockdown and equivalent doses of STDCONMO were injected for each experiment.

### TAG-1 mRNA rescue construct overexpression

To create a TAG-1 construct potentially capable of rescuing TAG-1 knockdown, we subcloned full-length TAG-1 into the *pCS2*^+ ^vector and then removed the first 20 bases of the sequence complementary to the 25-mer TAG1MO sequence, located in the 5' untranslated region. The mMessage mMachine Kit (Ambion, Austin, TX, USA) was used to transcribe 5' capped mRNA from *pCS2*^+^*-TAG1*. Approximately 100 pg of RNA was injected into one-cell stage embryos. Overexpression was verified by immunolabeling for TAG-1 at 24 hpf.

### Imaging

All brightfield images were captured on a Nikon TE300 inverted microscope equipped with a Spot RT camera (Diagnostic Instruments, Sterling Heights, MI, USA) and processed with Metamorph software (Universal Imaging Corp., West Chester, PA, USA). Fluorescent images of fixed preparations are confocal images captured on an Olympus Fluoview 1000 Laser Scanning Confocal Microscope.

### *In vivo *time-lapse imaging

Preparation of embryos for imaging was adapted from Langenberg and colleagues [54]. Briefly, the yolk cell was paralyzed by adenosine 5' (β,γ-imido)triphosphate (Calbiochem, San Diego, CA, USA) injection at 15.5 hpf, and then removed. Each embryo was mounted between two coverslips separated by high vacuum silicon grease in 67% L-15. Embryos ranged in age from 16–18 hpf at the start of imaging and were imaged for 2–12 hours in a temperature controlled environment set to 29°C. Images were captured using a 60× dipping objective (NA = 1.00) on a Nikon (Tokyo, Japan) E-600FN equipped with standard epifluorescence, a filter wheel, and a CoolSnap HQ camera (PhotoMetrics, Tucson, AZ, USA). Images were captured every one minute, and exposure times were typically 300–600 ms. Images and movies were captured, processed, and analyzed with MetaMorph software (Molecular Devices, Sunnyvale, CA, USA.

## Competing interests

The author(s) declare that they have no competing interests.

## Authors' contributions

MAW performed the experiments and data analysis, co-wrote the manuscript, and contributed to experimental design. VKS and AC designed, manufactured, and supplied the TAG-1 rescue construct, did *tag-1 in situ *hybridizations, and contributed to manuscript revisions. JJE and HJY cloned the *pitx2c *promoter, and designed and generated the *Tg(pitx2c:gfp) *transgenic line. MCH supervised the experiments, co-wrote the manuscript, and contributed to experimental design. All authors read and approved the final manuscript.

## Supplementary Material

Additional file 1 Time-lapse sequence of normal MLF axon outgrowth in an uninjected *Tg(pitx2c:gfp) *embryo. Ventral view, anterior to the left, ventral midline is up. The excerpt begins at 17 hpf. The movie shows MLF axons converging and MLF growth cones fasciculating along neighboring MLF axons. Images were captured every minute, and the movie runs at ten frames per second. Click here for file

Additional file 2 Time-lapse sequence of normal MLF axon outgrowth in an uninjected *Tg(pitx2c:gfp) *embryo. Ventral view, anterior to the left, ventral midline is up. The excerpt begins at 17 hpf. The movie shows a MLF growth cone (top left) extend into tissue outside the nucMLF zone, collapse, retract, and then fasciculate along an adjacent MLF axon. Images were captured every minute, and the movie runs at ten frames per second. Click here for file

Additional file 3 Time-lapse sequence of MLF axon outgrowth in a TAG1MO injected *Tg(pitx2c:gfp) *embryo. Ventral view, anterior to the left, ventral midline is up. The excerpt begins at 17 hpf. The movie shows MLF axons aberrantly growing into the tissue surrounding the nucMLF. Images were captured every minute, and the movie runs at ten frames per second. Click here for file

Additional file 4 Time-lapse sequence of MLF axon outgrowth in a *bal;Tg(pitx2c:gfp) *embryo. Ventral view, anterior to the left, ventral midline is up. The excerpt begins at 18 hpf. The movie shows MLF axons aberrantly innervating surrounding tissue and growing towards the ventral midline. Images were captured every minute, and the movie runs at ten frames per second. Click here for file

Additional file 5 Time-lapse sequence of MLF axon outgrowth in a TAG1MO injected *Tg(pitx2c:gfp) *embryo. Ventral view, anterior to the left, ventral midline is up. The excerpt begins at 17 hpf. The movie shows defective axon-axon interactions that precede MLF axon extension into surrounding tissues and alter neuronal polarity. Images were captured every minute, and the movie runs at ten frames per second. Click here for file

Additional file 6 Time-lapse sequence of MLF axon outgrowth in a *bal;Tg(pitx2c:gfp) *embryo. Ventral view, anterior to the left, ventral midline is up. The excerpt begins at 17.5 hpf. The movie shows MLF growth cones that maintain physical interactions with other MLF axons once contact is initiated. Images were captured every minute, and the movie runs at ten frames per second. Click here for file

Additional file 7 Time-lapse sequence of MLF axon outgrowth in a *bal;Tg(pitx2c:gfp) *embryo. Ventral view, anterior to the left, ventral midline is up. The excerpt begins at 18 hpf. The movie shows MLF growth cones that extend branches. Images were captured every minute, and the movie runs at ten frames per second. Click here for file
